# Toward more realistic career path prediction: evaluation and methods

**DOI:** 10.3389/fdata.2025.1564521

**Published:** 2025-08-25

**Authors:** Elena Senger, Yuri Campbell, Rob van der Goot, Barbara Plank

**Affiliations:** ^1^MaiNLP, Center for Information and Language Processing, LMU Munich, Munich, Germany; ^2^Fraunhofer Institute for Systems and Innovation Research ISI, Karlsruhe, Germany; ^3^Department of Computer Science, IT University of Copenhagen, Copenhagen, Denmark

**Keywords:** career path prediction, recommendation, synthetic data, LLM, labor market

## Abstract

Predicting career trajectories is a complex yet impactful task, offering significant benefits for personalized career counseling, recruitment optimization, and workforce planning. However, effective career path prediction (CPP) modeling faces challenges including highly variable career trajectories, free-text resume data, and limited publicly available benchmark datasets. In this study, we present a comprehensive comparative evaluation of CPP models—linear projection, multilayer perceptron (MLP), LSTM, and large language models (LLMs)—across multiple input settings and two recently introduced public datasets. Our contributions are threefold: (1) we propose novel model variants, including an MLP extension and a standardized LLM approach, (2) we systematically evaluate model performance across input types (titles only vs. title+description, standardized vs. free-text), and (3) we investigate the role of synthetic data and fine-tuning strategies in addressing data scarcity and improving model generalization. Additionally, we provide a detailed qualitative analysis of prediction behaviors across industries, career lengths, and transitions. Our findings establish new baselines, reveal the trade-offs of different modeling strategies, and offer practical insights for deploying CPP systems in real-world settings.

## 1 Introduction

Predicting career trajectories holds significant potential for both individuals and organizations. For professionals, accurate predictions can support informed career planning; for organizations, they enable better workforce development, recruitment, and retention strategies. Advances in NLP have enabled more sophisticated CPP methods, but selecting suitable models remains challenging especially in diverse, real-world contexts. Career Path Prediction aims to model this task by forecasting an individual's next likely occupation based on their career history. For example, given a sequence like *Software Engineer*→*Senior Engineer*→*Tech Lead*, a CPP model might predict roles such as *Engineering Manager* or *Principal Engineer*.

Historically, the lack of large, high-quality benchmark datasets has further hindered progress in this domain. Recent efforts have begun to address this, notably with the DECORTE dataset (Decorte et al., 2023) and the large-scale KARRIEREWEGE+ dataset (Senger et al., 2025). These datasets provide an essential foundation for evaluating and advancing career path prediction methodologies across diverse scenarios. In this paper, we present a comparative evaluation of career path prediction models across multiple real-world scenarios. We evaluate four major model types: (1) LSTM-based models, widely used in sequence prediction tasks, (2) a state-of-the-art linear projection baseline, (3) an MLP-based variant we introduce to increase expressiveness, and (4) a novel application of LLMs for standardized CPP–unexplored in prior work.

Beyond benchmarking, our goal is to provide practical insights for deploying CPP systems across diverse settings. We assess their performance in real-world settings, including scenarios with limited information, such as job titles as the sole input feature. We explore the impact of structured vs. unstructured inputs, by using standardized ESCO job titles and descriptions as well as free-text titles and descriptions. Furthermore, we analyze the impact of synthetic data and fine-tuning strategies on model performance. Additionally, we conduct a qualitative analysis of model predictions, examining trends across industries, job transitions, and career lengths.

**Our contributions can be summarized as follows:**

- A comprehensive comparison of CPP methods across multiple input settings and datasets, including novel model variants.
- Introduction of an MLP-based variant that achieves new state-of-the-art (SOTA) performance across several datasets and configurations.
- The first application of LLMs to standardized CPP, along with an analysis of grounding strategies and fine-tuning effects.
- An in-depth error analysis offering practical insights into model selection, optimization, and deployment in real-world CPP applications.
- A exploration of the role of synthetic data augmentation, highlighting quality and diversity considerations.

### 1.1 Related work

Career path prediction intersects multiple areas of research, including sequential modeling, occupational representation learning, and career trajectory analysis. In this section, we summarize key prior approaches to CPP, discuss recent advances involving LLMs, and position our work within this evolving landscape. We focus on both model architectures and input representations, highlighting where our contributions extend or adapt existing work.

#### 1.1.1 Career path prediction methods

CPP is a research area with a variety of methodologies. Some studies, such as (de Groot et al. 2021) and (Gugnani et al. 2018), utilize skill and occupation graphs to predict career trajectories. (Chang et al. 2019) introduced a Bayesian factorization model for predicting the next occupation. A widely adopted approach in this domain involves LSTM-based architectures (Li et al., 2017; Cerilla et al., 2023; He et al., 2022; Schellingerhout et al., 2022). The CAREER model (Vafa et al., 2024) leverages transformers to iteratively build representations of career histories using attention mechanisms across layers, showing strong performance when pre-trained on proprietary large-scale resume datasets and fine-tuned on smaller survey data. These advances reflect the growing role of language models in career prediction. (Decorte et al. 2023) used a fine-tuned sentence-transformer model to encode job titles, followed by a linear projection and skill-based scoring to represent career paths. More recently, (Du et al. 2024) introduced LABOR-LLM, which fine-tunes large language models (LLMs) on small survey datasets for next-job prediction, further improving on previous models like CAREER. LABOR-LLM generates free-text without being grounded in a label space.

Beyond model architectures, the input features used for prediction vary across studies. For example, (de Groot et al. 2021) integrate both job titles and skills into their models, while (Decorte et al. 2023) also incorporate detailed job descriptions. Other methods consider attributes such as years of education (Chang et al., 2019), as well as age, gender, and income (He et al., 2022), demonstrating the multifaceted nature of CPP research. We compare the effect of adding job descriptions to the occupation titles on the prediction accuracy.

#### 1.1.2 LLMs in recommendation

CPP shares structural similarities with sequential recommendation problems, as it involves predicting future roles based on a sequence of prior roles. This connection has led to our exploration of LLM-based approaches from recommendation systems to enhance CPP models.

A key challenge in applying LLMs in recommendation is the standardization of predictions within a predefined label space. Standardized prediction, grounded within a predefined label space, is often critical for effective evaluation and application. However, the existing CPP literature using LLMs overlooks this aspect. Recent works in the broader scope of LLMs for recommendation address these limitations by introducing *grounding mechanisms* for LLM outputs, i.e., methods to ensure that the model's generated sequences map back to valid item labels. As in (Geng et al. 2022), LLMs generate token sequences autoregressively using beam search, which are grounded to item identifiers through exact matching. Despite its utility, this approach can result in invalid item identifiers, necessitating additional matching strategies to map out-of-corpus recommendations to valid identifiers. To address the grounding challenge, (Bao et al. 2025) proposed a fine-tuning and an L2 distance matching method, leveraging again LLAMA to encode both generated sequences and the label space for grounding. Similarly, (Lin et al. 2024) employed fine-tuning for this end and implemented constrained generation techniques to ensure valid outputs.

In general, while fine-tuning helps in aligning unconstrained generations to a predefined label space, further post-training methods are necessary for full alignment. However, although constrained generation methods provide a useful framework, they rely heavily on the quality of initial tokens during beam search and are built upon the assumption of a unique label description or a known finite set of item descriptors (Lin et al., 2024). This dependency can limit their applicability in generating useful outputs for the CPP task. Hence, expanding on (Bao et al. 2025) and (Decorte et al. 2023), we compare grounding strategies for CPP based on dense representations of LLM generations.

### 1.2 Problem definition

The CPP task involves predicting the next occupation in a person's career trajectory based on their prior work history. A career history is represented as a sequential series of work experiences, each characterized by attributes such as job titles, descriptions, or skills. Unlike standard sequential recommendation tasks, CPP must handle non-standardized, free-text input data common in resumes, while managing the computational challenges posed by the diversity of possible transitions. On the other side, the sheer amount of possible free-form next experiences to be predicted would render the CPP task exceedingly challenging if not intractable. Most CPP methods rely on closed-source datasets, and there is a notable lack of publicly available datasets linked to standardized taxonomies (Senger et al., 2025). Such taxonomies are crucial for predicting standardized job titles, which are essential for many real-world applications, such as recommending job postings within specific job categories. To address this, we adopt the ESCO taxonomy^1^ as a structured classification system (Decorte et al., 2023). Formally, let a career history be a time-ordered sequence *C* = (*ex*_1_, …, *ex*_*N*_), where each *ex*_*i*_ is a work experience. The task is to rank ESCO occupation labels $E=\left\{occ_{1},\ldots ,occ_{\left|E\right|}\right\}$ by their suitability for the next experience *ex*_*N*+1_. Using ESCO version 1.1.2, |E| consists of 3,039 distinct labels. This approach ensures a balance between the flexibility of real-world data and the tractability of a structured label space, enabling effective career trajectory modeling.

## 2 Materials and methods

### 2.1 Datasets

Publicly available datasets for career path prediction are very limited (Senger et al., 2025). To the best of our knowledge, the only two publicly available datasets linked to a standardized taxonomy are DECORTE and KARRIEREWEGE+. This link is essential, as predicting standardized occupation titles (e.g., from ESCO) better reflects real-world applications and enables more robust evaluation.

The first dataset we use, DECORTE, is a small-scale dataset introduced by (Decorte et al. 2023). It was created using a Kaggle dataset of 2,482 anonymized English resumes, which were obtained by scraping individual resume examples from the livecareer.com website. However, it is unclear whether these resumes represent real-world data, as the origin of the individuals (e.g., their countries or professional situations) is unknown. The goal of livecareer.com is to assist users in improving their job search through tools such as resume enhancement, cover letter creation, and expert advice. (Decorte et al. 2023) linked all occupations in the dataset to ESCO (version 1.1.2) using a proprietary classifier. The dataset includes both self-written job titles and synthetic descriptions generated by summarizing the resume content.

The second dataset, KARRIEREWEGE+ (K+ ESCO) (Senger et al., 2025), is based on anonymized resumes provided by the German Employment Agency.^2^ This larger dataset contains 100,000 resumes of unemployed individuals in Germany, with standardized job titles from the German Berufenet taxonomy.^3^ These Berufenet titles were manually linked to ESCO (version 1.2.0). To enrich the data with free-text inputs, two data synthesis methods were employed. For version K+occ, LLAMA 3.1 8b was used to generate seven alternative titles for each ESCO occupation title. For K+cp, the entire sequence of career path titles was directly synthesized. Through quantitative and qualitative analyses, the authors found K+cp to produce higher-quality data. In this study, we examine which synthesis method works best for different models and analyze the influence of errors and patterns in the synthetic data on model performance. We use the official train/validation/test splits provided through Hugging Face for all datasets, which are approximately 80/10/10. Table 1 provides an overview of the different dataset versions used in this study, highlighting their respective sources, sizes, content types, and synthetization methods. Table 1 provides an overview of the different dataset versions used in this study, highlighting their respective sources, sizes, content types, and synthetization methods.

**Table 1:** Key characteristics of dataset versions used in our study.

**Dataset**	**Source**	**Country**	**Size**	**Titles**	**Descriptions**	**Synthetization method**
DECORTE	Kaggle / (livecareer.com)	Unknown	2,482	Self-written free text	Summarized by LLM	Summarization per work experience
DECORTE ESCO	Kaggle / (livecareer.com)	Unknown	2,482	ESCO titels	ESCO descriptions	–
KARRIEREWEGE+ occ	German Emp. Agency	Germany	100,000	Paraphrased by LLM	Paraphrased by LLM	Paraphrased 7 titles per occupation
KARRIEREWEGE+ cp	German Emp. Agency	Germany	100,000	Paraphrased by LLM	Paraphrased by LLM	Paraphrased per career path
KARRIEREWEGE+ ESCO	German Emp. Agency	Germany	100,000	ESCO titels	ESCO descriptions	–

### 2.2 Methods

We evaluate four distinct approaches across two practical scenarios: predicting the next occupation based solely on job titles and predicting the next occupation using both job titles and detailed job descriptions. These approaches are designed to handle both standardized and free-text inputs, ensuring applicability across datasets of varying sizes and complexities. Specifically, we conduct experiments on the DECORTE and KARRIEREWEGE datasets, which differ significantly in terms of size and contextual diversity, to ensure robust and generalizable findings.

Our selection of methods includes:

1. A recurrent LSTM model, widely used in CPP research and serving as a strong sequential baseline (Li et al., 2017; Cerilla et al., 2023; He et al., 2022; Schellingerhout et al., 2022).
2. A linear transformation baseline (Decorte et al., 2023), which achieved state-of-the-art performance in prior work.
3. A MLP model, proposed in this study as an extension of the linear baseline.
4. A LLM-based approach developed by us using Meta-Llama-3-8B-Instruct, drawing from recent advances in recommendation systems.

The LSTM and linear transformation approaches serve as strong baselines given their established performance in prior studies. Our MLP model, developed as an extension of the linear approach, introduces non-linear transformations to better capture complex patterns in career path data. Additionally, our LLM approach investigates the potential of large language models for standardized predictions, a previously unexplored area. To improve model performance across all architectures, we adopt the representation learning approach from (Decorte et al. 2023), which provides pretrained text embeddings for job titles and descriptions. The impact of this component is further analyzed in Section 3.2. This comparison allows us to systematically evaluate the performance and robustness of each model under diverse CPP scenarios, providing insights into their practical applicability and limitations. For evaluation we use Rean Reciprocal Rank (MRR), recall@5 (R@5) and recall@10 (R@10). All datasets,^4^ fine-tuned models ^5^ and code^6^ used are made available.

#### 2.2.1 Representation learning

**Figure 1:**
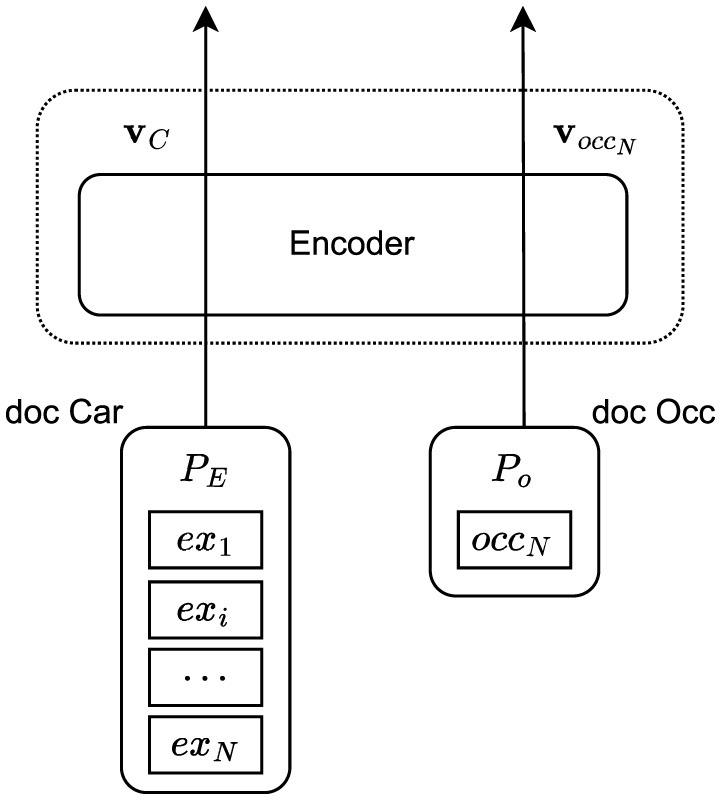
Representation learning stage, adapted from (Decorte et al. 2023).

Figure 1 illustrates the representation learning module. Here we follow (Decorte et al. 2023) and take the architecture and fine-tuning approach called “LAST”. This approach fine-tunes the all-mpnet-base-v2^7^ sentence-transformer model using contrastive representation learning on pairs of documents. Originally in (Decorte et al. 2023), each work experience *ex*_*i*_ in a career history is represented as:

role: <self-written title>

description: <synthetic description>

In our adaptation for the datasets with standardized (i.e. ESCO labels) input data, the career experience *ex*_*i*_ is represented as:

esco role: <esco occupation title>

description: <esco occupation description>

Similarly, in the setting with job titles as solely input, we represent the career experience *ex*_*i*_ as:

role: <esco occupation title>

or

esco role: <esco occupation title>

In all cases, the corresponding experiences are concatenated with a separator token “ < SEP>” to form a single career document, denoted as *doc_car*. The occupation document, denoted as *doc_occ*, is in turn structured always as the latter and contains data from only one ESCO occupation, here explicitly denoted as *occ*_*N*_, which corresponds to the last experience *ex*_*N*_, this being either self-written or ESCO data.

As shown in Figure 1, the model encodes the career history (*doc_car*) and the last ESCO occupation (*doc_occ*) into two embedding vectors: **v**_*C*_ and **v**_*oc*_*c*__*N*__. This encoding is achieved by an encoder trained with a Multiple Negatives Ranking Loss (MNRL) and in-batch negatives. We enhance the robustness of the representations by augmenting the career history data with all possible sub-histories of minimum length 2, also used in (Decorte et al. 2023). For example, given an original career history (*ex*_1_, *ex*_2_, *ex*_3_), the augmented histories include (*ex*_1_, *ex*_2_), (*ex*_2_, *ex*_3_), (*ex*_1_, *ex*_2_, *ex*_3_). This augmentation is applied after the data split. Fine-tuning is conducted using a batch size of 16, a learning rate of 2·10^−5^, and MNRL. Training runs for up to 1 epoch for the large (KARRIEREWEGE+) and two epochs for the small (DECORTE) datasets, with evaluation every 1% of steps based on validation loss. The best-performing model is saved based on these evaluations.

#### 2.2.2 LSTM

Given the sequential nature of career paths, we also frame the task of CPP as a time-series problem, where Long Short-Term Memory (LSTM) networks can be particularly effective. In this approach, the LSTM module takes as input the ordered sequence of experiences representations, ${\text{v}}_{C}^{1},{\text{v}}_{C}^{2},\cdots \;,{\text{v}}_{C}^{N-1}$, where ${\text{v}}_{C}^{i}$ is the representation from experience *ex*_*i*_. These embeddings are processed through bidirectional LSTM layers to capture the temporal dependencies and sequential patterns inherent in career trajectories.

We explore two main variants of LSTM architectures. The first variant incorporates an attention mechanism combined with global average pooling, inspired by the work of (Cerilla et al. 2023), to dynamically prioritize the most relevant parts of the input sequence. The second variant omits the attention mechanism entirely, instead relying solely on the final outputs from bidirectional LSTM layers to make predictions. Despite the absence of an attention layer, this simpler attention-free variant consistently outperformed the attention-based version across multiple experiments. Consequently, in the following result section, we report and compare results only for the variant without attention. For both architecture variants, we set the number of units in the bidirectional LSTM layers to 16, with a dropout of 0.1 and recurrent dropout of 0.2. The batch size was fixed at 16, and the learning rate was 0.001 using the RMSprop optimizer. We used categorical cross-entropy as the loss function, with early stopping based on validation loss and a patience of 10 epochs.

The output of the LSTM module is a probability distribution over the ESCO occupation set. This probability distribution is then directly used to rank the ESCO occupations, providing a prediction for the next likely occupation based on the given career history.

#### 2.2.3 Linear transformation

Following (Decorte et al. 2023), given an embedding model **E**, a linear transformation *T* is trained between career history embeddings $\text{E}\left(doc\text{\_}car^{1:N-1}\right)={\text{v}}_{C}^{1:N-1}$ and ESCO occupation embeddings **E**(*doc*_*occ*) = **v**_*oc*_*c*__*N*__ using the least squares method and no independent term. This transformation allows for a better alignment between the vector representations of career paths and their corresponding next ESCO occupation. Consequently, the prediction scores used to rank the ESCO occupation set are obtained by computing the cosine similarity between the transformed career path vector $T\left({\text{v}}_{C}^{1:N-1}\right)$ and all ESCO occupation vector representations.

#### 2.2.4 MLP

As an alternative approach to the linear transformation, we propose a Multi-Layer Perceptron (MLP) to align the career history embeddings with the next ESCO occupation embeddings. The MLP is trained using Cosine Embedding Loss (CEL) with stochastic gradient descent, aiming to maximize the cosine similarity between the transformed truncated career document representation $\text{MLP}\left({\text{v}}_{C}^{1:N-1}\right)$ and the actual next ESCO occupation document representation **v**_*oc*_*c*__*N*__. Key hyperparameters include a batch size of 16, a learning rate of 2·10^−5^, and a hidden layer configuration of 512 units. We train the model for up to 10 epochs with early stopping after 2 epochs of no improvement. Dropout is optionally applied with a rate of 0.1 to prevent overfitting. We experimented also with deeper MLP networks, however, they tended to overfit faster and resulted in lower performance. The prediction method remains the same as in the linear case. Hence, the choice for the CEL seems more natural, given the cosine-similarity-based scoring.

#### 2.2.5 LLM-based approach

##### 2.2.5.1 Fine-tuning

In this section, we describe the fine-tuning of the Meta-Llama-3-8B-Instruct model to enhance its ability to predict career paths based on structured inputs. This process involves adapting the pre-trained model using domain-specific datasets to better align it with the task of CPP. The fine-tuning was designed to optimize the model for both understanding structured instructions and generating relevant predictions. Previous work has shown that these steps also enhance the grounding in the label space so that the model generates items from the label space (Bao et al., 2025; Lin et al., 2024). This process leverages instruction tuning, adapting the model to respond to prompts that specify career histories and request predictions for subsequent roles. The fine-tuning approach employs the chat-based format illustrated below.

**System:** “*You are a job recommendation assistant trained to help users understand potential career paths based on their work history.”*

**User:**

“*Here is my work history:*

*1. Marketing Coordinator*

*2. Associate Product Manager*

*What would be the most suitable next role for me?”*

**Assistant:**

“*Category Manager”*

To standardize the input and output format, we utilized a predefined chat template, that structures the dialogue by marking roles, user inputs, and model outputs with special tokens. This template ensured that the model received and processed prompts in a standardized format, including special tokens to demarcate roles, user inputs, and model outputs. These formatted inputs improve the model's ability to generalize across different contexts while following instructions effectively. As in Section 2.2.1 the occupation histories (*ex*_1_, ⋯ , *ex*_*N*_) and the target occupation *occ*_*N*_ consist either of job title and descriptions or just the job title. The input career histories can additionally be in a free-text or standardized form.

We conduct standard supervised fine-tuning on the instructed model checkpoint LLAMA^8^ with cross-entropy loss function on token predictions. We choose to train on the full data point (prompt and completion) instead of masking the prompt in the loss function. We configured the tokenizer with a maximum sequence length of 2.048 tokens and right-side padding. Additionally, we set the AdamW optimizer to use a learning rate of 2·10^−4^ and gradient checkpointing to reduce memory usage during training, with an effective batch size of 2. The fine-tuning process used the SFTTrainer class for 1 to 3 epochs, depending on the dataset size. Moreover, we use Flash Attention 2 for optimized memory usage and processing speed. Fine-tuned models were periodically evaluated on a validation set, and the respective best-performing model checkpoint was saved based on the lowest validation loss. Fine-tuning of the LLAMA model demonstrates its adaptability to instruction-based tasks and its ability to provide accurate and contextually relevant career recommendations.

##### 2.2.5.2 Prediction and grounding

In prior studies such as (Lin et al. 2024), grounding strategies like L2 or cosine distance have shown limitations, particularly when the semantic similarity between generated tokens and valid identifiers is low. Unlike these cases, our domain–job titles and descriptions–is rich in semantic information, allowing more precise semantic matching. We propose a classification-based approach using a fine-tuned LLaMA-Instruct model to predict the next ESCO occupation. The method combines generative capabilities with embedding-based ranking for robust prediction.

Our approach uses a two-step pipeline inspired by (Bao et al. 2025), combining fine-tuning with L2 distance-based grounding. However, we enhance the pipeline by incorporating instruction tuning for improved alignment and replacing the LLAMA model with a specialized encoding model for embedding both label space and generated outputs. Given a career history, the fine-tuned LLaMA-Instruct model generates the next ESCO occupation using structured prompts and beam search. To ground predictions, the outputs and the label space are embedded using a fine-tuned all-mpnet-base-v2 model (see Section 2.2.1). L2 distance is then used to rank labels by proximity to the generated embeddings. This hybrid approach combines the interpretability of generative predictions with the robustness of embedding alignment.

Additionally, we experimented with extracting embeddings directly from the last hidden layer of the LLaMA model and comparing them to embedded labels (as well embedded with LLAMA). This approach avoids the intermediate generative step but showed significantly lower performance, underscoring the importance of the generative step in enhancing prediction quality.

## 3 Results

### 3.1 Comparison of models

Several broad trends emerge from the results shown in Table 2. First, the *KARRIEREWEGE+* datasets generally and unsurprisingly yield higher scores across most models than the DECORTE datasets, which is attributed to the positive impact of a larger training data size. Furthermore, *K+ESCO* outperforms the free-text datasets, possibly because of the low generation quality of the free-texts. This confirms that using free-text to replicate a real-world scenario is more challenging, where data is more diverse and less structured. In this context, free-text also serves to augment the data, making the model more robust. The robustness does not imply improved quality and in this case, it results in a trade-off in quality. Another plausible explanation is that a more standardized format facilitates pattern learning, particularly for the LSTM (which achieves the highest score here). This also offers an explanation why *K+occ* has slightly higher scores than K+cp, even though (Senger et al. 2025) showed that K+cp tends to have higher-quality synthetic free-text generations. The difference arises because K+occ is more standardized (only seven paraphrased titles per occupation), whereas K+cp has individually paraphrased career paths. In contrast, for the DECORTE datasets, the free-text variant seems to benefit from additional information not captured in the standardized version, yielding higher scores overall. For DECORTE, the *MLP* model achieves the highest MRR (25.57), whereas on DECORTE ESCO the best model varies between *linear, MLP* and *LLM* depending on the evaluation method. Meanwhile, *LSTM* shows weaker performance on DECORTE and DECORTE ESCO, likely because these datasets are too small for the LSTM to fully learn patterns. For the two K+occ datasets, *MLP* tends to perform best (e.g., MRR = 43.58 on K+occ, 43.02 on K+cp).

**Table 2:** Comparison of various models on multiple datasets.

**Model**	**DECORTE**			**DECORTE ESCO**			**K+ESCO**			**K+occ**			**K+cp**		
	**MRR**	**R@5**	**R@10**	**MRR**	**R@5**	**R@10**	**MRR**	**R@5**	**R@10**	**MRR**	**R@5**	**R@10**	**MRR**	**R@5**	**R@10**
**LSTM**	15.25	19.92	27.80	11.38	14.59	18.49	**49.13**	**60.96**	64.91	34.40	44.33	54.68	33.55	44.03	54.34
*only titles*	13.47	17.04	23.03	13.14	17.48	25.69	48.52	60.37	**65.01**	34.35	44.39	54.96	33.22	43.44	54.39
**Linear**	21.51	29.08	36.07	**20.84**	28.14	34.18	47.75	56.71	63.17	40.56	47.64	55.51	41.06	47.22	53.41
*only titles*	22.02	30.97	38.57	20.23	27.86	33.79	38.87	42.95	45.86	38.04	44.28	49.99	38.91	48.24	55.09
**MLP (ours)**	**25.57**	**35.13**	**42.56**	20.74	27.47	33.74	47.72	56.76	63.13	**43.58**	**53.38**	**61.77**	**43.02**	**52.71**	**60.37**
*only titles*	21.90	31.69	39.51	20.15	**29.74**	34.79	38.94	43.00	45.84	38.92	44.65	50.23	39.23	48.23	55.11
**LLM (ours)**	18.17*	28.91*	38.68*	17.49*	26.64*	35.13*	40.62	42.98	48.22	34.16	36.01	44.74	29.94	32.17	37.30
*only titles*	16.69*	26.19*	38.68*	17.90*	27.69*	37.57*	40.47	43.02	48.09	33.65	35.36	44.57	24.03	25.39	29.85

For each model, the top row reports results using both job titles and descriptions as input, while the “only titles” row shows results using only occupation titles. Evaluation metrics include Mean Reciprocal Rank (MRR), Recall@5 (R@5), and Recall@10 (R@10). Bold values indicate the best performance per dataset and metric, while underlined values represent the second-best. The star indicates, that the vanilla LLAMA model was used since it outperformed the fine-tuned version in these cases.

A key question is how relying solely on job titles, rather than including additional description fields, affects performance. As expected, in most cases, the “only titles” variants show a decrease in MRR, R@5, and R@10, indicating that extra textual context improves the models' ability to rank. For example, in K+ESCO, *linear* drops from an MRR of 47.75 to 38.87, while *MLP* similarly falls from 47.72 to 38.94 when only job titles are used. Interestingly, results on DECORTE show a milder trend or even a small improvement, e.g. for *linear only titles* (MRR = 22.02) compared to the full configuration (MRR = 21.51). However, in most cases the approaches lose accuracy when stripped down to job titles only, emphasizing the importance of extended textual features for these architectures.

Model-Specific Observations:

- **LSTM**: Lags behind on DECORTE and DECORTE ESCO but excels on K+ESCO, likely due to the LSTM's effectiveness with larger text. When restricted to only job titles, its performance deteriorates notably.
- **Linear**: Yields consistent performance, with particular strength on K+ESCO and the K+occ datasets. In most cases, the *only titles* configuration leads to moderate performance declines, though DECORTE shows a slight improvement.
- **MLP**: Often achieves the best or near-best metrics across multiple datasets (notably DECORTE, K+occ, and K+cp), suggesting it effectively leverages richer textual context.
- **LLM**: This approach does achieve top or near-top results on some scores for certain datasets, however, in most cases, it is outperformed by the other traditional approaches.

The relatively weak performance of LLMs in CPP tasks, despite large-scale pretraining, suggests that other elements such as fine-tuning strategies and decoding methods may play a more critical role. The main reasons likely lie in the training data and our fine-tuning strategy, which we discuss in detail in Section 3.2. Furthermore, our sampling strategy, greedy decoding, leads to predictions of poorer quality, as other LLM answers are not explored in the completions' space. When using beam search, the results for the LLM approach improve by increasing the number of beams, e.g for K+cp expanding the number of beams to 4 increases the MRR from 29.94 to 32.51, R@5 from 32.17 to 34.70 and R@10 from 37.30 to 39.82. Experiments with a higher number of beams were not done for all datasets due to computational restrictions. While beam search improves quality, it slows down inference significantly which made it impractical for larger test sets like KARRIEREWEGE+ (Zhang et al., 2023). Another open question is whether next-token prediction is the most suitable choice for the CPP task. While LLMs are flexible thanks to their natural language inputs, the sequential nature of CPP might benefit from other levels of granularity in the sequence space. This leads to the issue of grounding in the recommendation space (prediction space). The recommendation space grounding problem is well known in the literature of LLM-based recommendation systems and has, up to now, no conclusive solution (Wu et al., 2024). Following (Bao et al. 2025), we use a similarity-based grounding, which means retrieving candidates from the label space. This approach has limits as our retriever model (all-mpnet-base-v2, vanilla or fine-tuned) is not trained jointly with the LLM. This likely causes a mismatch between their tasks and limits overall performance in CPP.

In summary, the choice of model and the availability of detailed descriptions both significantly affect the ranking performance. The best approach partly depends on the dataset's size and characteristics: for example, *LSTM* excels at K+ESCO, whereas *MLP* is more robust across DECORTE and the *K+ free* datasets. Overall, the findings suggest that additional descriptive information typically leads to better results, although the extent of improvement varies by model and dataset.

### 3.2 Fine-tuning analysis

Table 3 highlights the influence of fine-tuning the all-mpnet-base-v2 model on different prediction approaches. Fine-tuning text representations significantly improves results for the MLP model, with performance gains of up to 10 percentage points. This improvement likely stems from the MLP's strong reliance on the quality of input representations, as it lacks the capacity to independently extract complex features from embeddings. The linear models benefit also substantially from fine-tuning since they are entirely dependent on the input representations to capture relevant patterns. In contrast, the LSTM approach benefits less from fine-tuning compared to the linear and MLP models. This is plausible since the LSTM's performance is influenced not only by the embeddings but also by its sequential modeling and the architecture of the classification head. Which demonstrates clearly the overall higher capacity of LSTM models, which, in this case can take advantage of the amount of available training data in order to find better representations during training when using a vanilla all-mpnet-base-v2.

**Table 3:** Influence of fine-tuning all-mpnet-base-v2.

**Model**	**DECORTE**			**DECORTE ESCO**			**K+ ESCO**			**K+occ**			**K+cp**		
	**MRR**	**R@5**	**R@10**	**MRR**	**R@5**	**R@10**	**MRR**	**R@5**	**R@10**	**MRR**	**R@5**	**R@10**	**MRR**	**R@5**	**R@10**
no ft LSTM	11.20	14.37	19.76	12.09	15.98	22.81	47.85	60.44	65.18	33.58	43.26	53.78	33.74	42.90	53.70
*Delta (ft - no ft)*	**+2.27**	**+2.67**	**+3.27**	**+1.03**	**+1.50**	**+2.88**	**+1.28**	**+0.52**	**-0.27**	**+0.77**	**+1.13**	**+1.18**	**-0.52**	**+0.54**	**+0.69**
ft LSTM	**13.47**	**17.04**	**23.03**	**13.14**	**17.48**	**25.69**	49.13	60.96	64.91	**34.35**	**44.39**	**54.96**	33.22	**43.44**	**54.39**
no ft linear	19.89	27.80	35.52	18.65	26.58	30.74	42.73	50.99	55.89	37.03	45.15	50.77	36.43	45.63	51.80
*Delta (ft - no ft)*	**+1.62**	**+1.28**	**+0.55**	**+2.19**	**+1.56**	**+3.44**	**+5.02**	**+5.72**	**+7.28**	**+3.53**	**+2.49**	**+4.74**	**+4.63**	**+1.59**	**+1.61**
ft linear	**21.51**	**29.08**	**36.07**	**20.84**	**28.14**	**34.18**	**47.75**	**56.71**	**63.17**	**40.56**	**47.64**	**55.51**	**41.06**	**47.22**	**53.41**
no ft MLP	17.69	27.08	34.79	19.35	25.58	32.08	42.65	50.72	55.59	38.32	46.15	51.41	37.52	46.76	52.60
*Delta (ft - no ft)*	**+7.88**	**+8.05**	**+7.77**	**+1.39**	**+1.89**	**+1.66**	**+5.07**	**+6.04**	**+7.54**	**+5.26**	**+7.23**	**+10.36**	**+5.50**	**+5.95**	**+7.77**
ft MLP	**25.57**	**35.13**	**42.56**	**20.74**	**27.47**	**33.74**	**47.72**	**56.76**	**63.13**	**43.58**	**53.38**	**61.77**	**43.02**	**52.71**	**60.37**

Bold values indicate the best performance per dataset and metric.

The results presented in Table 4 demonstrate the benefits of fine-tuning both the generation model (LLAMA) and the embedding model (all-mpnet-base-v2) on the KARRIEREWEGE+ datasets. Specifically, the fine-tuned LLAMA model paired with the fine-tuned all-mpnet-base-v2 achieves the best performance across both datasets, as highlighted by the scores from the “Checkpoint 2 epochs” configuration. Interestingly, the performance benefits of using the fine-tuned all-mpnet-base-v2 are only realized when the LLAMA model is also fine-tuned. This suggests that the fine-tuned all-mpnet-base-v2 relies on the specific format of titles and descriptions it was trained on and which are also generated by the fine-tuned LLAMA model. These are absent in the vanilla LLAMA outputs. Moreover, Table 4 reveals that employing LLAMA as a post-generation encoder, as proposed by (Bao et al. 2025), substantially degraded performance in our experimental setting. One possible explanation for these results is the dataset structure in our use case, which entails longer texts per item. Additionally, while LLAMA excels at generation tasks, its specialization for embedding tasks is limited compared to all-mpnet-base-v2, which is explicitly optimized for encoding tasks. Overall, these results highlight the importance of aligning the generation and retrieval models through fine-tuning for optimal performance. The findings also emphasize the need for selecting task-specific models: LLAMA's strength lies in text generation, while all-mpnet-base-v2 is more adept at encoding.

**Table 4:** Results for K+occ only titles and K+ESCO only titles dataset samples (1,000 data points) for various settings predicted with the LLM approach.

**Model**	**K+occ only titles**			**K+ESCO only titles**		
	**MRR**	**R@5**	**R@10**	**MRR**	**R@5**	**R@10**
Checkpoint 1 epoch	23.56	23.30	25.30	35.26	35.70	37.60
Checkpoint 1 epoch, non-fine-tuned mpnet	23.08	23.00	23.80	35.01	35.20	36.40
Checkpoint 2 epochs	**30.23**	**30.40**	**33.00**	**36.38**	**36.60**	**38.80**
Checkpoint 2 epochs, non-fine-tuned mpnet	29.93	29.70	32.00	35.81	35.90	37.50
Vanilla generation	14.67	19.30	23.80	16.66	20.20	24.90
Vanilla generation, non-fine-tuned mpnet	16.13	18.00	24.10	29.07	33.40	38.30
Vanilla generation, LLAMA embeddings	1.04	0.50	1.50	1.18	0.60	2.80

The checkpoints indicate the length of the LLAMA fine-tuning “no fine-tuned mpnet” means vanilla all-mpnet-base-v2 was used for the retrieval of the label space. “vanilla generation” means that the LLAMA model was not fine-tuned and “LLAMA embeddings” that LLAMA was used for retrieval instead of all-mpnet-base-v2. Bold values indicate the best performance per dataset and metric.

Table 5 highlights the performance of different configurations of the LLM approach on the DECORTE dataset. The results consistently show that the vanilla model outperforms the fine-tuned versions across all metrics. In combination with Table 4, this suggests that LLM fine-tuning has a mixed effect in the CPP task. The amount of training data clearly plays a critical role in performance outcomes. Our experiments demonstrate that even with minimal fine-tuning in the DECORTE dataset (one epoch) performance drops significantly compared to the vanilla variant. As fine-tuning continues with limited data, overfitting becomes more apparent, further reducing effectiveness. This finding raises questions about the optimal data volume required for CPP in low-resource scenarios. Additionally to overfitting another plausible explanation for the superior performance of the vanilla model is its tendency to generate a broader list of potential next occupations, while fine-tuned models focus on a single occupations they are trained to do. This broader output may inadvertently enhance the retrieval process, leading to better overall results. However, Table 4 indicates that under different data conditions, LLM fine-tuning of up to two epochs can still benefit the CPP task. Nevertheless, performance consistently peaks and then declines beyond after two epochs. Overall, LLM performance fall behind alternative models across all examined checkpoints, indicating persistent limitations even before reaching the overfitting regime.These results suggest a misalignment between next-token prediction and the nature of the CPP task. The low diversity and repetitive structure of CPP data, which often contains many repeated career paths, likely contribute to this issue. Consequently, a different sampling strategy within the available data might yield improvements for LLM-based CPP approaches.

**Table 5:** Results for various settings of the LLM approach on the DECORTE dataset.

**Model**	**MRR**	**R@5**	**R@10**
Checkpoint 3 epochs	01.8	01.9	02.9
Checkpoint 1.3 epochs	11.27	12.0	22.0
Checkpoint 1 epoch	13.96	18.0	27.0
Vanilla generation	**18.2**	**28.9**	**38.7**

Bold values indicate the best performance per dataset and metric.

In conclusion, under given data and decoding settings, LLMs show clear limitations for CPP. In low-resource environments, vanilla models outperform fine-tuned variants, suggesting that conventional fine-tuning approaches may actually impair performance in this setting. While limited fine-tuning shows improvements under certain data conditions, a consistent performance ceiling exists for LLM-based approaches. This points to a fundamental mismatch between standard LLM training and the demands of career path prediction.

### 3.3 Error analysis and subset performance

In this section, we analyze how the different approaches perform on specific subsets of the data. We include ESCO industries with more than 50 test data points, as well as long career paths (those with more than five former occupations) and short career paths (fewer than three former occupations). To ensure a fair comparison of long and short career paths, we further filter each to exclude those samples in which the last occupation is the same as the ground truth–this situation is often found in short subsequences and tends to be easier to predict. We also examine career changes, defined as cases where the target occupation is in a different major ESCO category than the last occupation in the career history. This career change does not necessarily mean that the industry changes, for example “pastry maker” is in “Craft and related Trades Workers”, whereas “pastry chef” part of “Technicians and associate professionals”. Additionally, we look at progressions, in which the last occupation is not a “Manager” but the target occupation is, and regressions, where the last occupation is part of the ESCO category “Manager” but the target is not.

**Figure 2:**
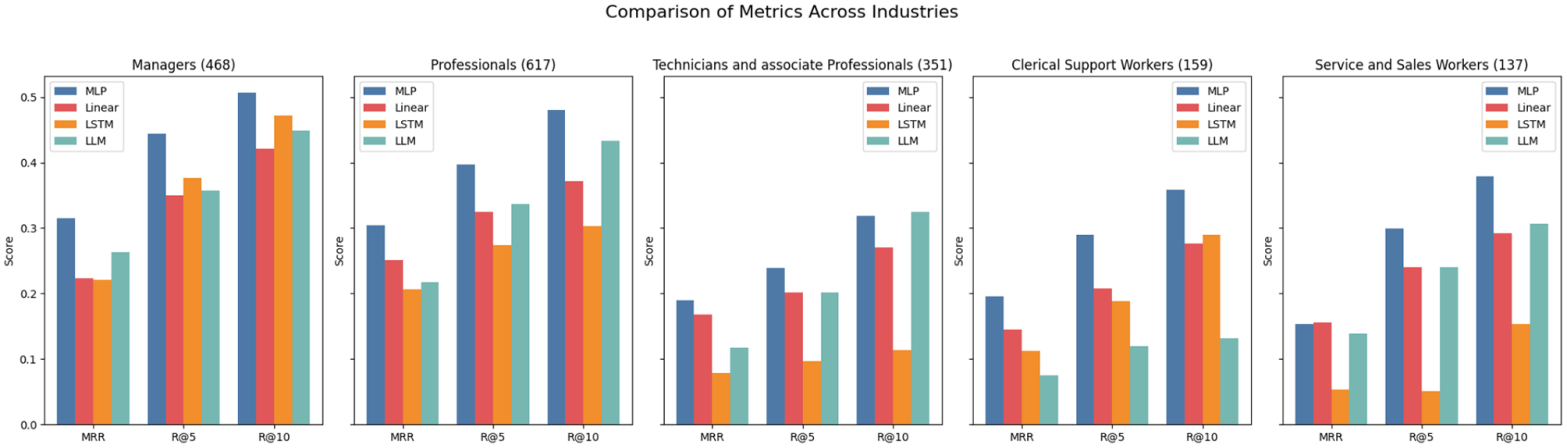
Metrics for DECORTE across ESCO categories. The numbers in the brackets indicate the number of data points available for the subset.

**Figure 3:**
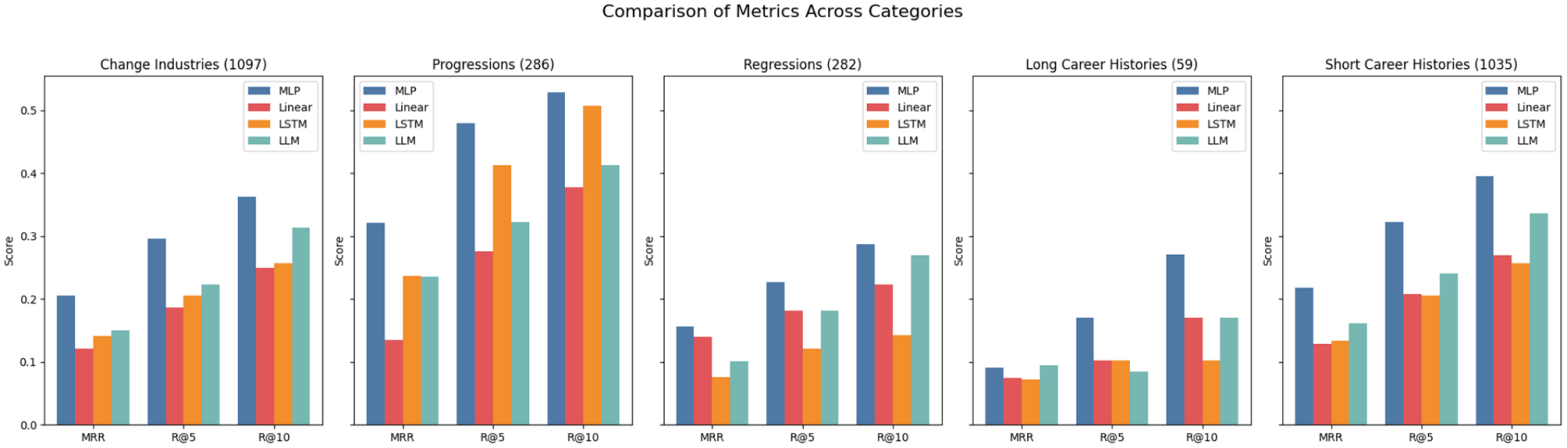
Metrics for DECORTE across subsets. The numbers in the brackets indicate the number of data points available for the subset.

Interestingly, in the DECORTE dataset, certain approaches perform much better at predicting specific occupational categories (see Figure 2). Examples are the LLM approach, which performs well for all categories but the “Clerical Support Workers” category, or the LSTM performing well for “Managers” but poorly for “Service and Sales workers”. All models show very high scores for the“Managers” and “Professionals” ESCO categories with the highest number of data points, indicating a positive effect of more train data. Additionally, we can see in Figure 3, that it is generally easier to predict progressions than regressions, which makes sense if progressions appear more often in the data or align more closely with the LLAMA model's pretraining data. Contrary to expectations, longer career paths do not necessarily improve prediction accuracy; in fact, shorter paths produce higher scores. This might be because longer paths often involve more industry switches, making it harder to predict whether someone will revert to a former occupation. Notably, for the DECORTE dataset, the MLP outperforms all other approaches for all subsets in each metric.

**Figure 4:**
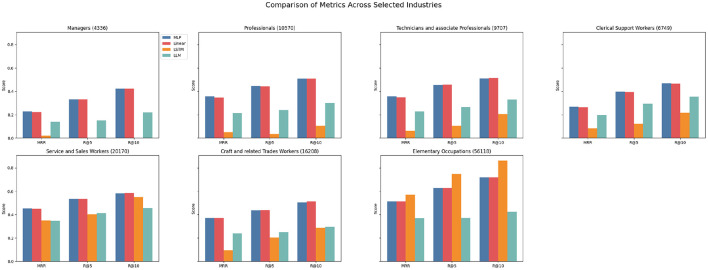
Metrics for K+cp across ESCO categories. The numbers in the brackets indicate the number of data points available for the subset.

**Figure 5:**
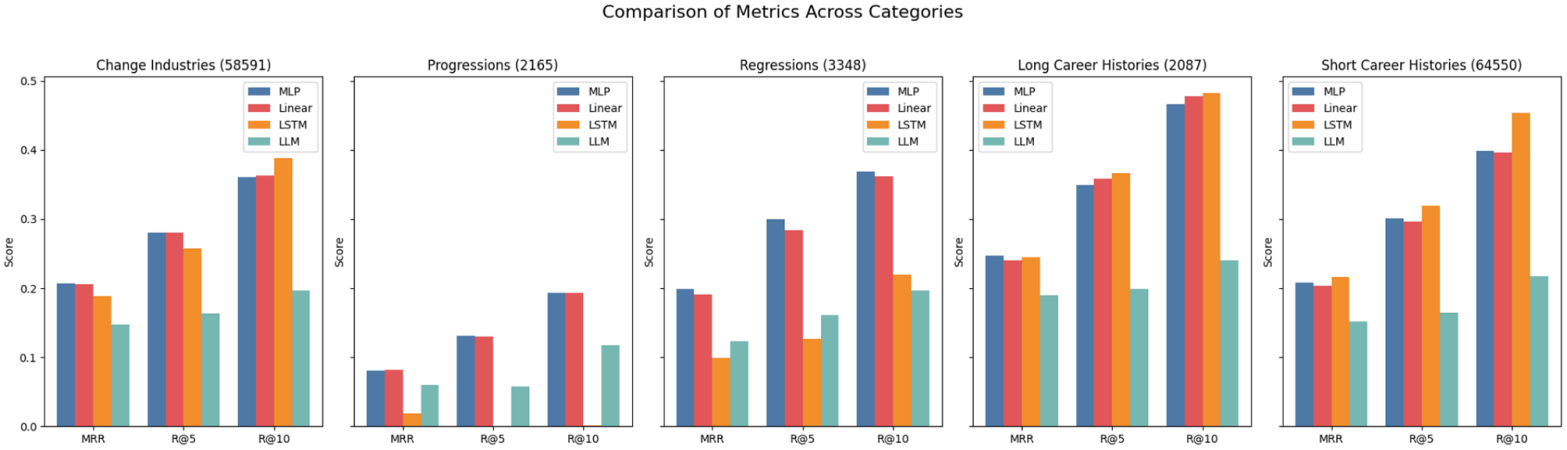
Metrics for K+cp across subsets. The numbers in the brackets indicate the number of data points available for the subset.

**Figure 6:**
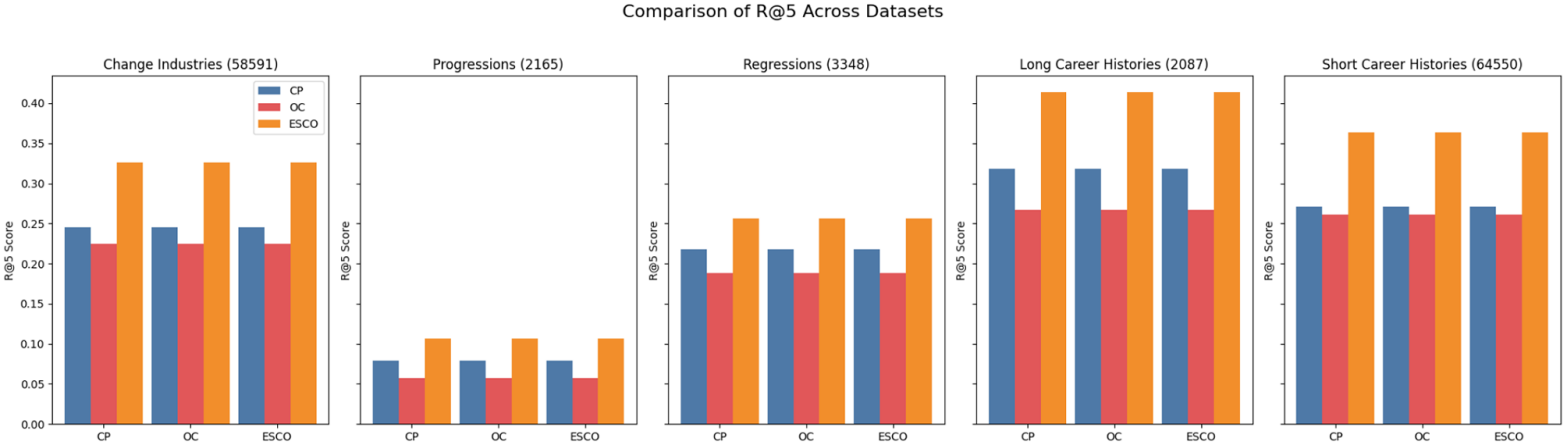
Metrics for KARRIEREWEGE+ datasets across subsets. The scores are the mean across all approaches for R@5. The numbers in the brackets indicate the number of data points available for the subset.

Across all three KARRIEREWEGE+ datasets and prediction approaches, the scores for the ESCO categories “Armed Forces Occupations”, “Skilled Agricultural, Forestry, and Fishery Workers” and “Plant and Machine Operators and Assemblers” were consistently close to zero. The first two categories are the ones with the fewest data points, which could explain the low performance. Compared to DECORTE, the predictive quality for “Managers” is relatively lower than other ESCO categories. This is expected, given that the share of data points for this category in the dataset is smaller (see Figure 4). Conversely, the“Elementary Occupations” and “Service and Sales Workers” categories exhibit strong performance, likely due to their high representation in the dataset. The MLP and linear approaches yielded the best results across all categories, except for “Elementary Occupations” which is the most represented category. Here, the LSTM demonstrated strong performance, likely because its architecture effectively learned the prevalent patterns in this category. Figure 5 further highlights the robustness of the MLP and linear approaches. However, while the LSTM performs well for longer career histories, it struggles to accurately predict progressions or regressions. Interestingly, as shown in Figure 6, the K+cp approach outperforms K+occ across all subsets, even though K+occ achieves higher overall results. This trend remains consistent for MRR and R@10.

**Figure 7:**
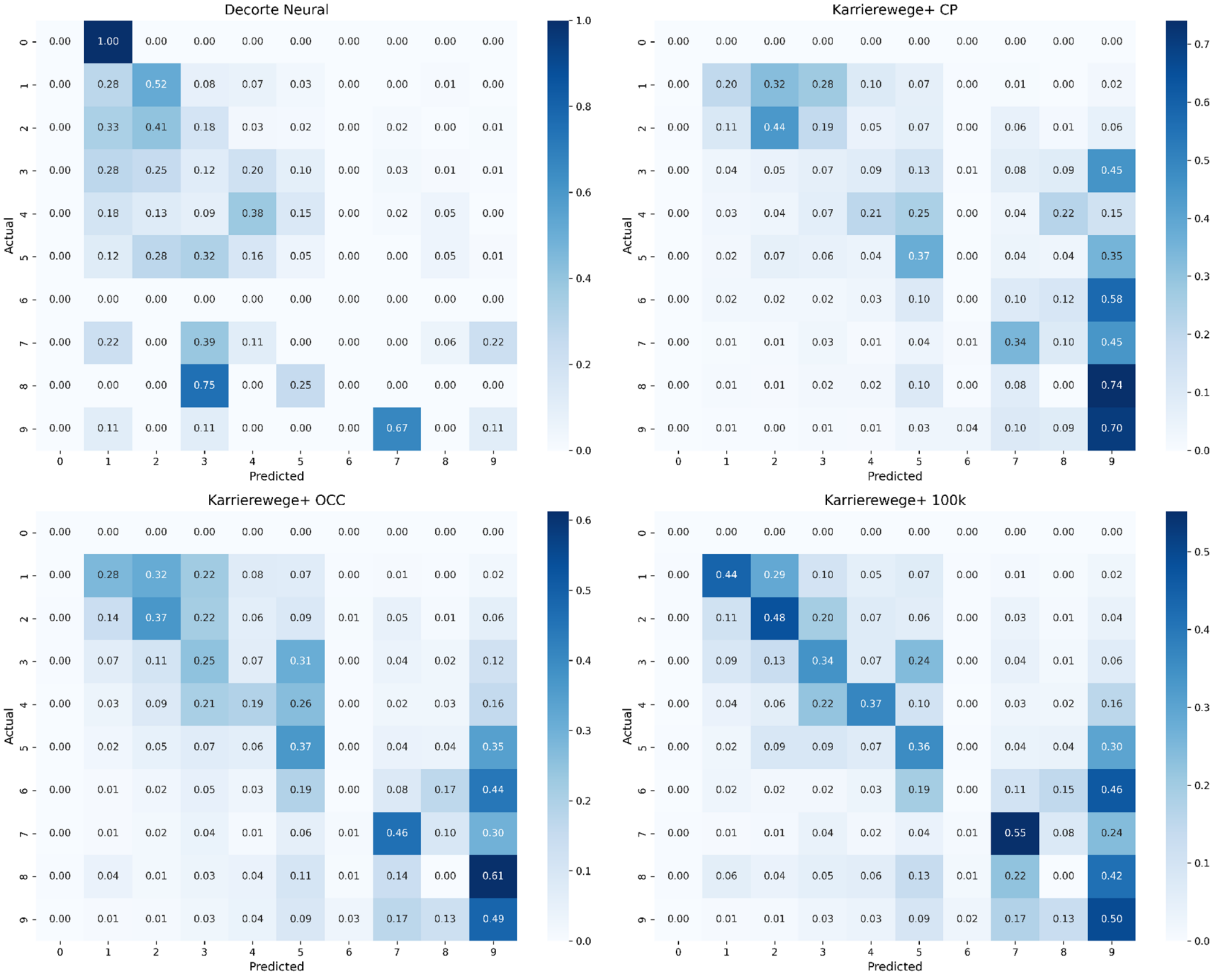
Normalized top-1 confusion matrices for the MLP approach across four datasets. Values represent row-wise proportions.

**Figure 8:**
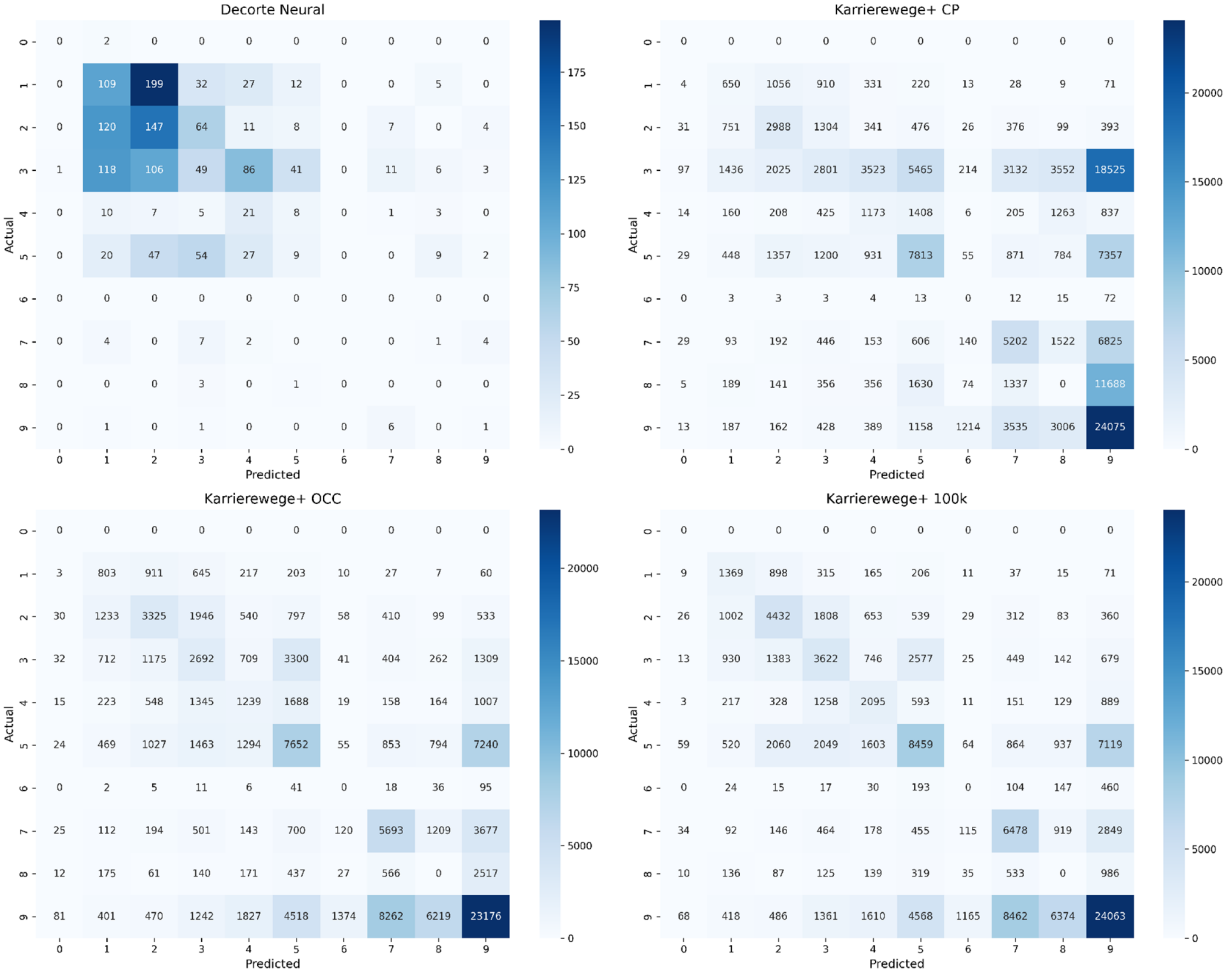
Absolute top-1 confusion matrices for the MLP approach across four datasets. Values represent raw prediction counts.

To complement the subset and category-level analyses, we include confusion matrices based on top-1 predictions for the MLP approach across the four datasets (see Figures 7, 8). These matrices provide a closer look at where the model makes specific misclassifications among first-level ESCO categories (see Table 6 for code mappings). The normalized confusion matrices (Figure 7) show the proportion of predictions per true label. They reveal frequent confusion between categories like 5 (Service and Sales Workers), 7 (Craft and Related Trades Workers), and 9 (Elementary Occupations), especially in the KARRIEREWEGE+ datasets. In contrast, the absolute matrices (Figure 8) reflect the data imbalance–categories with more samples, such as 9, dominate predictions, while rare classes like 0 and 6 are underrepresented in predictions.

**Table 6:** First level of the ESCO classification.

**Code**	**First level occupation category**
0	Armed forces occupations
1	Managers
2	Professionals
3	Technicians and associate professionals
4	Clerical support workers
5	Service and sales workers
6	Skilled agricultural, forestry and fishery workers
7	Craft and related trades workers
8	Plant and machine operators and assemblers
9	Elementary occupations

### 3.4 Synthetic data as error source

For the K+occ and K+cp models, we conducted a qualitative analysis to evaluate whether there is an indication that synthetic data serves as a significant source of error. Specifically, we manually examined 100 random samples of incorrect predictions made by K+occ and K+cp (using the MLP approach with job title and description as input). In these cases, we assessed how often errors in the synthetic titles or descriptions occurred in the incorrectly predicted data samples. To provide a baseline, we compared these results with the quality of 100 random synthetic titles and descriptions evaluated in (Senger et al. 2025).

This comparison revealed a pattern of error propagation: the mean correctness score of synthetic text dropped from 4.72 to 3.76 for K+occ and from 4.82 to 4.02 for K+cp in the error cases. In other words, the quality of synthetic input was substantially lower in the subset of failed predictions than in the overall sample. This indicates that the presence of errors in synthetic job titles and descriptions is not random, but correlates with—and likely contributes to—model failure.

In addition, we observed that in 37% of K+occ cases and 35% of K+cp cases, the ground truth label corresponded to the same ESCO occupation as the model's previous prediction, yet the model still failed to predict correctly. This suggests that the synthetic input distorted an otherwise straightforward prediction. In other instances, there was a logical progression within the ESCO occupation framework, but this transition was obscured due to poor-quality generation–making it harder for the model to infer the correct occupation.

The example career paths provided in Table 7 illustrate common error patterns in synthetic career paths. For instance, in cases labeled under “Too Specific Occupations”, synthetic job titles often deviated from generalizable terms, leading to mismatches with the ground truth. For example, the synthetic title “Geotechnical Specialist” was generated for a worker described as a “Civil Engineering Worker”, which introduced unnecessary specificity and caused prediction errors. Similarly, in the “Semantic Mismatch” category, errors arose when job titles were paraphrased in a way that altered their meaning or misrepresented logical career progressions. An example of this can be seen with the ground truth “Foster Care Support Worker”, where the paraphrased title “Case Management Coordinator” misaligned with the intended role, leading to inaccurate predictions. In the “Increasing Professionalism” category, the synthetic paraphrasing added professional-sounding job titles, such as changing “Mover” to “Logistics Coordinator”. While this might sound more sophisticated, it created inconsistencies that disrupted the logical connection to the ground truth. Finally, in the “Not Specific Enough” category, the synthetic job titles became overly generic, obscuring key information necessary for accurate predictions. For example, the ground truth “Radio Technician” was paraphrased as “Electronics Specialist”, which generalized the role too broadly and led to a mismatch.

**Table 7:** Categorization of common error patterns in synthetic career path predictions →, with examples of career paths of various length.

**Category**	**Original job titles**	**Paraphrased job titles**
**Increasing professionalism**	Mover, wood caulker → mover	Logistics coordinator, building sealant technician → mover
	Domestic cleaner → domestic cleaner	Housekeeping manager → Domestic cleaner
**Too specific occupations**	Civil engineering worker building construction worker civil engineering worker → civil engineering worker	Geotechnical specialist site worker urban planner assistant → civil engineering worker
	Doorman/doorwoman → doorman/doorwoman	Nightclub host/hostess → doorman/doorwoman
**Not specific enough**	Garden laborer crop production worker → crop production worker	Cultivator agricultural specialist → crop production worker
	Radio technician → radio technician	Electronics specialist → radio technician
**Semantic mismatch**	Social work assistant → foster care support worker	Case management coordinator → foster care support worker
	House sitter → house sitter	Home care provider → house sitter

These examples illustrate that synthetic errors–whether due to over-specificity, semantic shifts, inflated professionalism, or excessive generalization–mislead the model and degrade performance. A more comprehensive error analysis is available in (Senger et al. 2025).

## 4 Discussion

### 4.1 Conclusion

This study presents a comprehensive evaluation of methods for CPP, exploring both established and newly introduced model architectures, including linear models, MLPs, LSTMs, and LLMs. Leveraging two benchmark datasets, DECORTE and KARRIEREWEGE+, we investigated the impact of data size, structured vs. unstructured inputs, limited information (using only titles), and synthetic data quality on model performance. Our findings underscore the importance of data representation and distribution in determining model effectiveness. Categories with limited data points, such as *Armed Forces Occupations* and *Skilled Agricultural, Forestry, and Fishery Workers*, consistently showed poor predictive performance. Models relying on job titles and descriptions generally outperformed those using only job titles, emphasizing the importance of textual context in capturing nuanced career trajectories. Among the methods evaluated, the newly introduced MLP model demonstrated robustness across multiple scenarios and archived new SOTA prediction scores. LSTM models, while excelling in specific settings like long career histories, struggled with less represented subsets, as well as small datasets, due to their sequential nature. LLM-based approaches exhibited potential, particularly when grounded within structured label spaces, but their performance was highly dependent on fine-tuning and alignment with the dataset. A key insight from this research is the trade-off between synthetic data augmentation and prediction quality. While synthetic data enhances model robustness by diversifying inputs, enables fine-tuning of LLMs without overfitting, and facilitates the effective use of LSTMs, errors in its generation–such as overly specific or overly generic paraphrasing–can adversely affect performance.

### 4.2 Further research

The findings of this study open several avenues for future research in the domain of CPP. First, addressing data scarcity in underrepresented categories is critical. Techniques such as data augmentation, transfer learning, and domain-specific pre-training could be employed to enhance predictive accuracy in these categories. Second, the application of advanced grounding strategies in LLM-based approaches could improve alignment with structured label spaces. Techniques such as constrained generation may enhance prediction accuracy by mitigating the challenges posed by unstructured inputs. Finally, future work should focus on improving the quality and diversity of synthetic data generation. By identifying and mitigating common error patterns–such as semantic mismatches and excessive generalization–it is possible to enhance the reliability of models trained on augmented datasets. In conclusion, this study establishes a strong foundation for advancing CPP methodologies, offering actionable insights into model selection, optimization, and real-world applicability. By addressing the challenges outlined, future research can drive the development of more accurate, equitable, and context-aware systems for predicting career trajectories.

## Data Availability

The original contributions presented in the study are included in the article/supplementary material, further inquiries can be directed to the corresponding author.

## Footnotes

^1^ https://esco.ec.europa.eu/en/about-esco/what-esco

^2^ https://www.arbeitsagentur.de/bewerberboerse/

^3^ https://web.arbeitsagentur.de/berufenet/

^4^KARRIEREWEGE+: https://huggingface.co/datasets/ElenaSenger/Karrierewege_plus, DECORTE: https://huggingface.co/datasets/TechWolf/anonymous-working-histories

^5^ https://huggingface.co/collections/ElenaSenger/karrierewege-67c5bdd7d60a81183b28079d

^6^ https://github.com/elenasenger/realistic-career-path-prediction

^7^ https://huggingface.co/sentence-transformers/all-mpnet-base-v2

^8^ https://huggingface.co/meta-llama/Meta-Llama-3-8B-Instruct
